# Fungal Biostarter Effect on the Quality of Dry-Aged Beef

**DOI:** 10.3390/foods12061330

**Published:** 2023-03-21

**Authors:** Wiesław Przybylski, Danuta Jaworska, Magdalena Płecha, Karina Dukaczewska, Grzegorz Ostrowski, Piotr Sałek, Krzysztof Sawicki, Julia Pawłowska

**Affiliations:** 1 Department of Food Gastronomy and Food Hygiene, Institute of Human Nutrition Sciences, Warsaw University of Life Sciences (WULS), Nowoursynowska 166, 02-787 Warsaw, Poland; 2 Institute of Evolutionary Biology, Faculty of Biology, Biological and Chemical Research Centre, University of Warsaw, Zwirki i Wigury 101, 02-089 Warsaw, Poland; 3 CHRIS, Dry-Ager, Szwedzka 23/75, 30-324 Kraków, Poland

**Keywords:** beef aging, sensory quality, *Mucor flavus*, microbiology of meat, biostarters

## Abstract

Meat aging is a process consisting of its storage in specific conditions which leads to an increase in its organoleptic qualities. The most common method of meat aging is the wet vacuum-bag based method, whereas the traditional method, called dry-aging, involves keeping meat at a low temperature for an extended time. However, this process is characterized by low repeatability of the results. Therefore, different approaches to stabilize the process are tested. The aim of this study was to analyze the influence of the *Mucor flavus* biostarter on the physicochemical characteristics and sensory quality of dry-aged beef (DAB). We hypothesized that a fungal biostarter positively influences the quality of DAB and stabilizes the dry-aging process. Meat control samples (N = 7) and samples inoculated with the *Mucor flavus* biostarter (N = 7), originating from 14 individuals crossbred from Holstein-Friesian cows with bulls of meat breeds, were put into the dry-aging fridge (DryAger, Bad Saulgau, Germany) for 28 days. The physicochemical parameters (pH, color parameters, WHC, GP (glycolytic potential), chemical composition of muscle, the content of malondialdehyde, shear force), muscular protein proteolysis (SDS-PAGE), sensory quality, and microbial composition of DAB were assessed after aging. The results showed a significant effect of the fungal biostarter on pH increase (0.25 units), and light myosin chain proteolysis (approximately 16%) as well as improvement of sensory quality (e.g., acceptability was improved by one unit in an applied scale 1–9). All together, the *M. flavus*-based biostarter was shown to increase the quality of DAB.

## 1. Introduction

Beef is the third most-consumed meat in the world after poultry and pork. This meat can be an important source of high-value protein, rich in essential amino acids, heme iron with high bioavailability, zinc, and B vitamins [1]. However, buying beef, consumers are guided not only by its nutritional value, but also by its sensory quality [1].

For beef, much attention has focused on intramuscular fat level, color, tenderness, juiciness, and flavor. Color and intramuscular fat are the first characteristics perceived by the consumers and they are often the only two traits that are considered at the time of purchasing of packaged meat. However, tenderness is the most crucial sensory quality and can be defined as the ease with which meat can be sliced or chewed [1]. This trait is influenced by many factors and, therefore, is difficult to predict and evaluate. Juiciness represents the perception of meat as more or less dry during chewing. The flavor of meat is the combined result of two senses, taste and aroma, in an olfactory–gustatory perception [1]. These qualities of beef are developed by many breeding and nutritional factors and may be later influenced by changes taking place in the muscle tissue postmortem. Due to the importance of these characteristics, extensive research has been carried out to enable qualitative classification of beef and, based on those characteristics, to predict the perception of the meat’s quality by the customers [1,2,3,4].

One of the very important stages in developing the sensory quality of beef is its aging process after slaughter. Postmortem aging is a value-adding process and has been extensively practiced by the global meat industry for years. Various forms of aging are practiced, ranging from traditional carcass hanging to packaging sub-primals or portion cuts in vacuum bags for a certain duration of cooling storage [5]. Dry- and wet-aging are the two most common forms of postmortem aging used to enhance tenderness and aid in flavor development of beef products [6,7]. Wet-aging is more common and refers to meat aged in a vacuum-sealed bag filled with saline solution and stored in a controlled environment for a specific period of time. Dry-aging is the process of hanging beef carcasses, sub-primals, or unpackaged primal cuts in a refrigerated room or fridge and left to age for several weeks or even months at controlled temperature, relative humidity, and air flow with spontaneous microbiological occurrence [6,7]. The production of beef by dry-aging is expensive due to the large losses, which may be as high as 40%, associated with water evaporation and the need to remove discolored and dehydrated surface layers of muscle and fat tissue [6,7,8]. Beef produced this way is a niche product that meets the expectations of consumers who appreciate its unique sensory qualities such as taste or aroma [8,9]. Most often, this method of aging is used by small and medium manufacturers, and such beef is available in exclusive shops and luxury restaurants, as well as hotels, so-called steakhouses, specializing in serving ‘tender beef’ [8,9]. Some producers believe that this process can be considered as a kind of art and each of them usually has its own unique conditions of meat aging [8,9]. However, incorrect aging processes may lead to the growth of pathogenic microorganisms on the surface of the meat and significant loss of material [10,11]. Therefore, different methods seeking to stabilize the dry-aging process are investigated. The use of appropriate safe microorganisms’ cultures could be used to standardize the process for the general meat industry. Such biostarters are used, for example, in the production of ripened sausages [12]. Only recently, several studies focused on fungal diversity in DAB, e.g., [10,11] and its potential application in stabilizing the dry-aging process. Two species of fungi originally isolated from DAB, *Pilaria anomala* SMFM201611 (*Mucoromycota*) and *Debaryomyces hansenii* SMFM201707 (*Ascomycota*), were shown to improve the quality of dry-aged beef [13]. Similarly, Hangasaki and Asato [14] reported usage of Mucor flavus strain in the beef dry-aging process. The changes in the free amino acid content, hardness, productive loss, drip, and cooking loss were demonstrated for this strain. Mucor flavus strains are of particular interest as representatives of this species and were traditionally used in Asia for sufu (fermented soybean curd) production [15]. The species is known to be psychrotolerant, rapidly growing mucoralean fungus with proteolytic activity [16].

The aim of the research was to analyze the influence of another *M. flavus* strain on the physicochemical characteristics and sensory quality of dry-aged beef as well as its effect on DAB microbiome diversity. We hypothesized that the fungal biostarter positively influences the quality of DAB and stabilizes the dry-aging process.

## 2. Materials and Methods

### 2.1. Research Material

#### 2.1.1. Sample Collection and Preparation

The meat for the experiment originated from 14 individuals being crossbred from Holstein-Friesian cows with bulls of meat breeds (bred and slaughtered in the western region of Poland; Greater Poland). In order to standardize the material for research, the samples were taken from R class (taking into account the muscularity) and 2 class (considering the fatness) according to EUROP system of cattle classification after slaughter. The slaughterhouse was located in Greater Poland region. The animals were slaughtered in accordance with the European Union Council Regulations (EC) No. 1099/2009 for the protection of animals at the time of slaughter. After chilling of carcasses, the samples were taken from *Longissimus* muscle (Lumbar part)—as an entrecote culinary element and transported to the laboratory in chilling conditions. The average weight of samples was around 3.5 ± 0.5 kg.

#### 2.1.2. Fungal Biostarter

The fungal strain KKP 2092p from the culture collection of the University of Warsaw was used as biostarter in this experiment. The strain was isolated from dry-aged beef retrieved from a local Warsaw butcher in 2020. The strain was identified as *Mucor flavus* Bainier [MB#179990]. The fungal strain KKP 2092p is assigned under following application patent number: P.443722.

### 2.2. Research Methods

#### 2.2.1. Aging Conditions

The aging of meat was performed in a dry-aging fridge (DX 1000 Premium S, DryAger, Bad Saulgau, Germany) at temperature of 1.5 °C with approximately 80–90% relative humidity for 28 days. The pieces of beef (approx. 3.5 kg each) were put into the cabinet: the control samples (N = 7; henceforth called biostarter −) and the test samples with the previously applied *M. flavus* biostarter (N = 7; henceforth called biostarter +).

Fungal starter culture was grown on 4% Sabouraud Dextrose Agar (SDA) plates for 7 days in 21 °C and then removed from plates in order to be lyophilized. Lyophilized mycelium was then suspended in the Sabouraud broth (30 g of lyophilized mycelium in 1 L of broth) and kept in 21 °C for 24 h. For 1 kg of meat, 10 mL of prepared inoculum (10 mL/1 kg) was distributed on the test samples. The control samples were covered only with Sabouraud broth in the same manner and amount. The cuts of beef were placed in the dry-aging fridge and turned twice a week, to prevent the plasma and other fluids from accumulating in one part of the meat. After every 7 days of aging, the meat was weighed and the weight loss due to evaporation was assessed. The weight losses were calculated at 7, 14, and 28 days of aging as the percent of losses in relation to the initial weight. Temperature and humidity were monitored daily during the aging process.

#### 2.2.2. The pH Determination

The pH value [17] was measured (in triplicate) the day after slaughter (before aging) and after 28 days after slaughter (after aging) using the automatic pH meter 330 i (WTW^®^, Weilheim, Germany), equipped with special electrodes SenTix^®^ SP Number 103645, to measure pH directly in the meat. The electrode was inserted to a depth of 0.5 cm in the central parts of the meat slice. The pH meter was calibrated before measurements using standard phosphate buffers (pH 4 and pH 7).

#### 2.2.3. Color Measurement

Color parameters (CIE L*a*b*) were also measured the day after slaughter (before aging) and after 28 days of aging using a CR-310 Konica Minolta ^®^ Chroma Meter (Osaka, Japan) on the cross-section of each piece of meat in triplicate. Meat chops (5 × 5 cm) were cut and bloomed for 1 h at 4 °C with no surface covering prior to color measurements. The apparatus was calibrated before each measurement against a white and black tile. The color was expressed according to the Commission International de l’Eclairage (CIE) system and reported as L* (lightness), a* (redness), and b* (yellowness) with a D65 light source.

#### 2.2.4. Glucose and Lactate Concentration in Tissue, and Muscle Glycolytic Potential

Glucose and lactate concentrations were measured in drip loss by using an Accu-Chek Active^®^ glucometer (Accu-Chek Sensor Comfort^®^, Roche, Germany), as described by Przybylski et al. [18]. The muscle glycolytic potential (PG) was calculated according to the formula PG = (2 glucose) + lactate adapted from the work of Monin and Sellier [19] and was expressed as millimoles (mmol) of lactate.

#### 2.2.5. Water-Holding Capacity and Meat Plasticity

Water-holding capacity (WHC) known as filter paper press method as an indicator of free water content in meat was estimated according to Grau–Hamm method [20]. The procedure was as follows: a sample (in duplicate) of minced meat (3 mm), with a mass of 0.3 g, was placed on a filter paper with a specific sorption power (Whatman 1) between two glass plates and subjected to an even load with a 2 kg weight for 5 min. After drying, an image of blotting paper with the contours of the dripstone (free water) and flesh (plasticity) was obtained. The method of computer image analysis was used to measure the surface areas of the stains. WHC is expressed as the amount of cm^2^ of dripping per 1 g of meat, while the meat plasticity is expressed in cm^2^ of an area crushed on a tissue sample of meat.

#### 2.2.6. Meat Composition

Meat composition (water, fat, protein, collagen, and ash content) was determined using a near-infrared spectrometer NIRFlex N-500 (Büchi, Flawil, Switzerland). Measurements were conducted using a NIRFlex Solids module of spectral range 12,500–4000 cm^−1^ in reflectance mode. The values of standard error of calibration (SEC) for moisture, fat, protein, ash, and collagen were 0.33, 0.19, 0.27, 0.20, and 0.11, respectively, whereas standard error of cross-validation (SECV) was 0.28, 0.14, 0.27, 0.19, and 0.09, respectively. Coefficient of determination in calibration (R2) for moisture, fat, protein, ash, and collagen measurement was 0.91, 0.96, 0.97, 0.94, and 0.97, respectively. Meat samples weighing approx. 100 g were homogenized, placed on a glass dish (1 cm layer), and covered. Three measurements of each sample were conducted. During one measurement, each sample was scanned 32 times [17].

#### 2.2.7. Determination of the Shear Force

Determination of the cutting force was conducted using the ZWICKI 1120 apparatus (Zwick–Roell GmbH & Co. KG, Ulm, Germany). From the heat-treated meat samples, samples with a cross-section of 1 × 1 cm with a longitudinal arrangement of muscle fibers were cut (5 individual measurements). During the test, the maximum force of cutting the meat sample with a Warner–Bratzler attachment (equipped with a flat knife) and the depth of penetration of the knife at which the maximum force occurred were measured. An initial force was 0.5 N and the test speed was 50 mm/min. A measuring head with a range of 2–1000 N was used [17].

#### 2.2.8. The Content of Malondialdehyde

The level of lipid oxidation in aged meat after 28 days of aging was evaluated using the TBA method, based on the content of malondialdehyde (MDA) [21]. Two grams of minced meat and 5 cm^3^ 10% trichloroacetic acid (TCA) were put into a centrifuge tube. The mixture was subjected to intensive mixing. Next, 5 cm^3^ 0.02 M 2-tiobarbituric acid (TBA) solution was added to the mixture and again the content of the tube was mixed. Afterward, the tubes were centrifuged (4000 rpm during 10 min). After centrifuging, the content of the tubes was filtered into glass tubes. The tubes were covered with plastic foil and put in a boiling water bath for 35 min in order to develop the color. Simultaneously, the reagent test was prepared. The content of the tubes was cooled in cold water. After cooling, the absorbance was measured in solutions at a wavelength of λ = 532 nm in a spectrophotometer (Thermo Scientific, Waltham, MA, USA, Genesys 20). The results were expressed as the content of MDA in meat [mg/kg]. Each sample of meat was evaluated in triplicate.

#### 2.2.9. Sodium Dodecyl Sulfate–Polyacrylamide Gel Electrophoresis (SDS-PAGE)

The SDS-PAGE of muscular tissue was performed according to the method of Bollag and Edelstein [22] using the STANDARD system (Kucharczyk TE, Warsaw, Poland). Proteins were resolved on a 12% separation gel and 5% stacking gel. Myofibrillar proteins were extracted from 20 mg of muscle, homogenized with 800 μL of a Tris-HCl buffer (pH 6.8) containing 0.375 M 2-mercaptoethanol, 3% SDS, 8 M urea, and 2 M thiourea. The muscle protein concentration was determined as total nitrogen by the AOAC method (1). Extracted proteins were dissolved 1/1 (*v/v*) in a Tris-HCl sample buffer (pH 6.8) containing 0.375 M 2-mercaptoethanol, 3% SDS, 8 M urea, 2 M thiourea, and 0.05% bromophenol blue. The mixture was then heated for 3 min at 95 °C, and 10 μL of the sample was placed in each well. Gels were first run for approximately 1 h at 75 V followed by 5 h at 150 V. Gels were stained with Coomassie Brilliant Blue R250. Image analysis and quantification were performed with GelScan v. 1.45 software (Kucharczyk TE, Poland). Molecular weight markers (Fermentas International INC., Burlington, ON, Canada) were used to estimate the molecular weights of the proteins.

#### 2.2.10. Consumer Evaluation of the Sensory Quality of Dry-Aged Beef

The evaluation was carried out at the Institute of Human Nutrition Sciences, Warsaw University of Life Sciences (laboratories for food evaluation and food preparation). The consumers (N = 40) were invited to participate in the study through an advertisement at the university. The survey was voluntary among those who declare themselves as beef consumers.

The pieces of meat, steaks 2.0 cm thick (cut using automatic cutter Ma-Ga, 250 W; 50 Hz, Bydgoszcz, Poland), were set aside until they reached room temperature (20 °C), and grilled using a hot contact grill (PK2745E, 3000 W, 60 Hz, Potis GmbH, Goettingen, Germany) at 250 °C until medium-rare degree of doneness. Average time of grilling was 3 min.

The grilled steaks were set aside and 2.0 cm × 2.5 cm pieces were cut perpendicular to the muscle fibers for sensory evaluation. Meat samples were placed in odorless disposable plastic boxes with lids, which were identified by code numbers. Samples were separately coded for each assessment with three-digit codes and were served to an evaluation.

There were two stages in the consumer study. Stage A. Firstly, the aroma of raw steaks was assessed by each consumer. The set of 2 samples of steaks, control, without biostarter and sample with *M. flavus* biostarter, were presented to the consumers who evaluated the aroma liking (odor A) of steaks before heat temperature in a 1–9 point hedonic scale. Stage B. Then, the second step was conducted, and samples were assessed after grilling. The evaluated attributes were aroma (odor B), softness, juiciness, flavor, and overall liking. All participants rated each of the meat samples on a 9-point hedonic category scale with end anchors of 1—extremely disliked and 9—extremely liked [23].

Each participant received a sample separately coded with three-digit codes. The samples were served one by one to each consumer (divided in 4 groups). The assessment and the condition mode were determined in accordance with Meilgaard et al. [24] and Baryłko-Pikielna and Matuszewska [25].

The evaluation was conducted in a specially prepared stand during daylight illumination. Between evaluations, the consumers received water to neutralize the taste.

The study was conducted in accordance with the Helsinki Declaration (World Medical Association 2013) [26] and with the regulation and approval of the Ethical Commission No. 15/2021.

#### 2.2.11. Fungal Biostarter Taxonomic Identification

The taxonomic assignment of used fungal biostarter was performed based on molecular identification techniques. Total genomic DNA was extracted from pure fungal culture on 4% SDA medium using DNA extraction kit EM13 (Blirt S.A., Gdańsk, Poland) according to the manufacturer’s instructions. The internal transcribed spacer (ITS) fragment was amplified and sequenced as described by Siedlecki et al. [27]. Received sequences were compared with reference sequences deposited in GenBank database (www.ncbi.nlm.noh.gov).

#### 2.2.12. Bacterial DNA Extraction

Total genomic DNA was isolated from two pieces of dry-aged entrecote, maturated for 28 days. One of the pieces was aged with *M. flavus* biostarter, while the other without it. Two samples from each piece were selected for molecular analyses from two distant areas of studied meat pieces by cutting 10 g of surface tissue fragments. A total of four samples were homogenized in 90 mL of saline solution (0.9% NaCl) from which 1 mL was collected and centrifuged (10,000× *g*, 5 min) as previously described by Ryu et al. [25]. Pellet obtained after centrifugation was resuspended in 675 µL of CTAB solution for DNA extraction (EURx, Gdańsk, Poland), 25 µL proteinase K (20 mg/mL, EURx, Gdańsk, Poland) was added, and the solution was incubated in 55 °C for one hour [28]. Inactivation of any remaining enzymes was performed at 65 °C for 15 min. An amount of 700 µL of chloroform:isoamyl (24:1) was added to dissolve nonpolar impurities such as fatty acids. Solution was mixed gently by inverting tubes for 10 min and centrifuged for 5 min at 3000× *g*. Top layer was collected into the new tubes and 5 µL of RNAse (Blirt S.A., Gdańsk, Poland) was added, gently mixed, and incubated in 37 °C for 20 min. The chloroform:isoamyl mix (24:1) was then repeated. Top layer was transferred into 15 mL falcon tubes and 300 µL of 5 M NaCl solution, DNA solution, and 1.8 mL of 96% ethanol cooled to −80 °C were added. Samples were cooled down for one hour at −20 °C to facilitate DNA precipitation. Samples were centrifuged and the supernatant was discarded. Pellet was washed with 1 mL of 70% ethanol, centrifuged in 4 °C for 7 min at 3000× *g*, and the supernatant was discarded. Washing with ethanol solution was repeated once. Samples were left to dry overnight and were dissolved in DNAse-free water. Quality and quantity of DNA was assessed via agarose gel (1%) electrophoresis (1 × TAE buffer, 80 V, 45 min, room temperature) and spectrophotometrically with NanoPhotometer NP80 (Implen, Munich, Germany).

#### 2.2.13. Bacterial Metabarcoding

Bacterial communities of DAB were characterized using high-throughput sequencing (HTS) of 16S rDNA amplicons. For preparation of the V3–V4 regions of 16S rDNA amplicons, the following primer pair was used: 341F 5′-CCTACGGGNGGCWGCAG-3′ and 785R 5′-GACTACHVGGGTATCTAATCC-3′ [29]. Four amplicons (2 samples biostarter + and 2 biostarter −) were prepared using high-fidelity KAPA HiFi DNA Polymerase (Roche, Basel, Switzerland) and were sequenced on an Illumina MiSeq platform (Illumina, CA, USA) by the Genomed company (Warsaw, Poland) using a v3 MiSeq chemistry kit in the paired-end mode (read lengths 2 × 250 bp). Raw sequencing data were deposited in the National Center for Biotechnology Information’s (NCBI) Sequence Reads Archive (SRA) under the project number PRJNA858677.

Sequencing reads were quality controlled using FastQC [30]. Accepted 16S rDNA amplicons sequences were classified using Qiime2 [31] with dada2 pipeline and taxonomic assignment based on Naïve Bayes classifier trained on Silva database v. 138 as downloaded in April 2022. Taxonomic profiles were used to present the bacterial community composition. The alpha-diversity (Shannon, Chao1, and Simpson indexes) as well as beta-diversity (principal coordinates analysis, PCoA) were calculated using the phyloseq R version 1.22.3 package [32]. For plotting, ggplot2 R version 3.3.5 package [33] was used.

#### 2.2.14. Statistical Analysis

The basic descriptive statistics (mean, standard error of the mean) were calculated. The Shapiro–Wilk test was used for testing of normality of the distribution of data. The significance of differences between the groups in terms of the traits characterizing meat quality (biostarter—versus biostarter+) was determined using Student’s *t*-test. The effect of aging time and groups for weight loss of meat was evaluated by ANOVA. LSD (Last Significant Differences) test was applied for evaluation of significance of differences between means. The statistical significance coefficient *p* < 0.05 was used. The obtained data were calculated using STATISTICA version 13.3 software (TIBCO Software Inc. 2017, Palo Alto, CA, USA, Statistica data analysis software system, version 13, http://statistica.io, accessed on 28 July 2022).

## 3. Results

### 3.1. Physicochemical Changes of DAB

The data show that the groups did not differ significantly before dry-aging in terms of tested quality traits (Table 1). The fresh beef used for aging characterized a high quality, meaning that its pH value, color parameters, glycolytic potential, water-holding capacity, plasticity, and basic chemical composition were typical for normal and good-quality meat.

**Figure 1:**
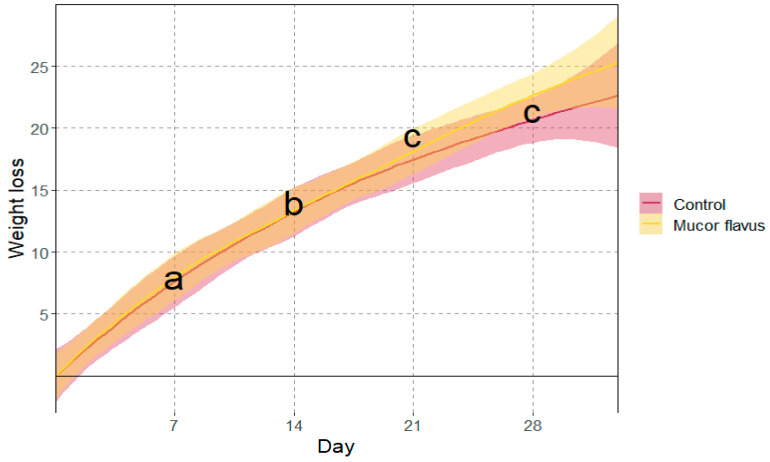
Weight loss of meat during dry-aging with and without biostarter. The orange and red shadows around lines stand for 95% confidence interval; a, b, c—means marked with different letters differ significantly at *p* < 0.05.

The analysis of variance showed that the weight loss of meat during aging significantly increased with time after 7, 14, and 21 days of aging. In contrast, the difference between the 21st and 28th day of aging was insignificant. Overall, the average meat weight losses during aging were 7.7% 13.8%, 19.4%, and 21.4%, after 7, 14, 21, and 28 days, respectively (Figure 1).

No significant differences in weight loss were found between the control samples and those after dry-aging with biostarter. No significant differences were found between the two studied groups in terms of the quality traits (Table 2). However, the samples aged with the *M. flavus* biostarter were characterized by a slightly lower shear force after grilling, a lower content of malondialdehyde, and a slightly higher pH value (Table 2).

**Figure 2:**
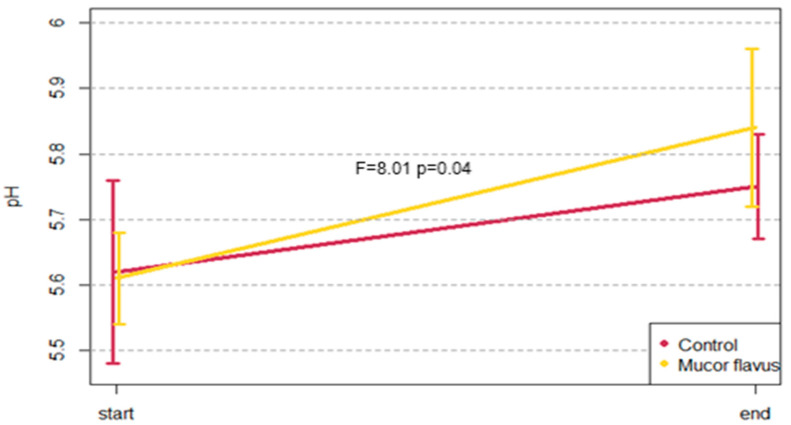
Changes of pH during aging. Average values with standard deviation bars are shown.

The analysis of variance showed that during the aging of entrecotes with the *M. flavus* biostarter, the pH increased significantly in comparison to control (Figure 2). The difference in pH value between both samples seasoned with *Mucor flavus* and control ones increased by 0.24 and 0.14 units, respectively (Table 1 and Table 2).

### 3.2. Myofibrillar Proteins Profiles of DAB

**Figure 3:**
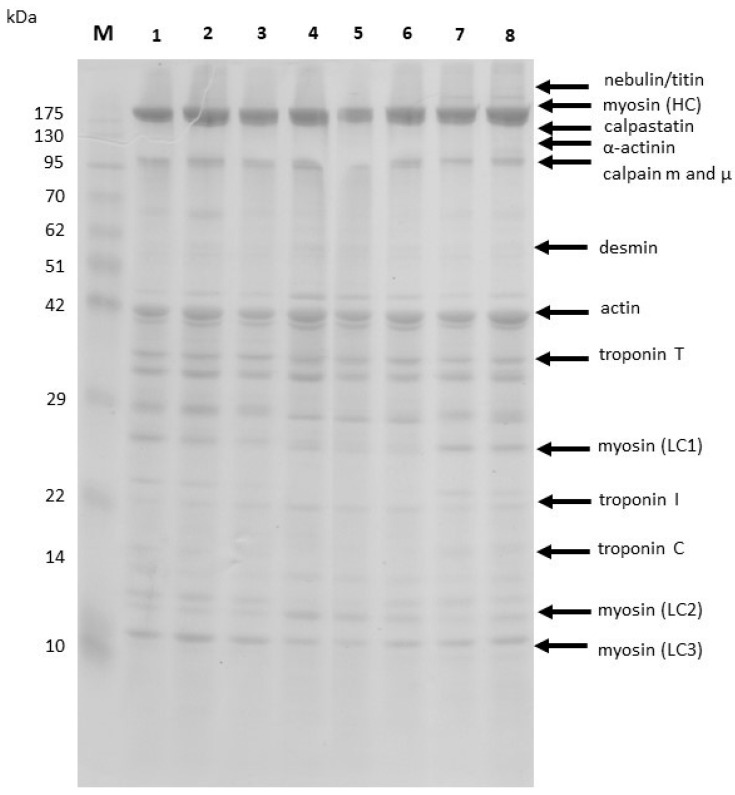
The SDS-PAGE profile of proteins from the aged meat samples; Lane M—marker, Lane 1—control (inside), Lane 2—control (outside), Lane 3—Mf (inside), Lane 4—Mf (outside), Lane 5—control (inside), Lane 6—control (outside), Lane 7—Mf (inside), Lane 8—Mf (outside).

**Figure 4:**
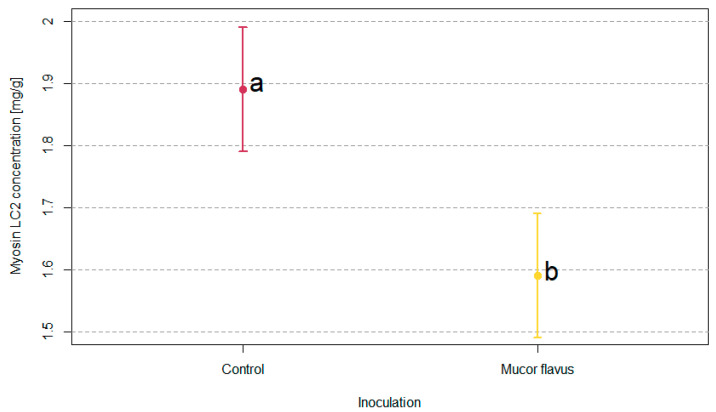
Differences in proteolysis of myosin LC2 chain. Average concentrations with standard deviation bars are shown; a, b—means marked with different letters differ significantly at *p* < 0.05.

SDS-PAGE of myofibrillar proteins was performed to assess the changes in the proteolytic profiles during the aging process (Figure 3). The meat samples for this analysis were derived from the external dry cuticle and internal layers of the aging pieces of meat. Only the number of LC2 myosin light chains proved to differ significantly in densitometric analysis between tested samples. Dry-aged beef pieces with the *M. flavus* biostarter were characterized by a significantly lower amount of LC2 myosin light chains (Figure 4).

### 3.3. Changes in Sensory Quality of Dry-Aged Beef

**Figure 5:**
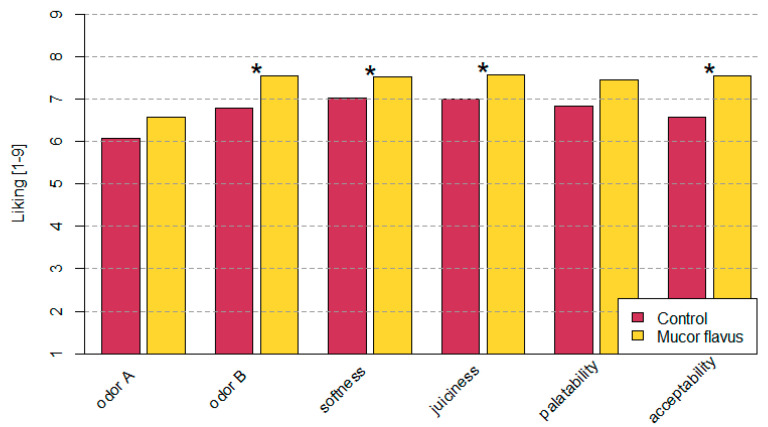
The results of consumer evaluation of the sensory quality of dry-aged beef. Significant differences according to Student’s *t*-test (at *p* < 0.05 are marked with *).

Significant differences were observed between the tested samples when looking at the following parameters: aroma of meat after heat treatment, softness, juiciness, and acceptability. More acceptable aroma after grilling was achieved by the steaks from the meat steaks aged with *M. flavus* biostarter addition. These steaks were not only softer and juicier but also their overall acceptability was much higher when compared to control stakes (Figure 5). The described differences were statistically significant.

### 3.4. Bacterial Diversity of DAB

From the Illumina MiSeq sequencing, 1,261,340 paired-end reads were obtained (rarefaction curves are given in Figure S1). The mean number of reads per sample was 157,668 (adapters were already removed by the sequencing company). Reads with Phred quality score less than 20 (Q20%) were discarded. After quality evaluation, which included denoising, length and quality trimming, and chimeric sequence exclusion, 410,178 sequences remained and were grouped into 37 ASVs.

The alpha-diversity indices (Chao1, Shannon, and Simpson) differ between the biostarter—and biostarter + samples, with higher species richness in control samples (Table 3). As the Chao richness estimator gives more weight to the low-abundance species [34], the higher species richness of control samples was more pronounced for this estimator. However, the differences in Shannon and Simpson measures between the two control samples were also observed. The bacterial species taxonomic diversity was not correlated with pH changes, color parameters changes, or shear force after grilling (Table 3).

**Figure 6:**
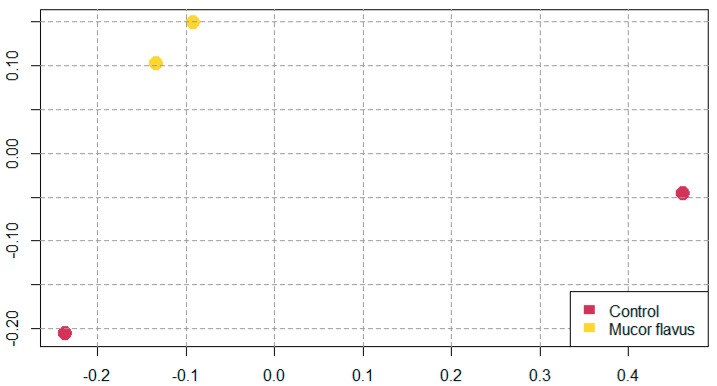
PCoA plot based on Bray–Curtis dissimilarity measure showing the variation of ASV communities from different meat samples.

Principal coordinates analysis confirmed that samples aged with the *M. flavus* biostarter are microbiologically similar, whereas control samples form distinct and more variable bacterial communities (Figure 6).

**Figure 7:**
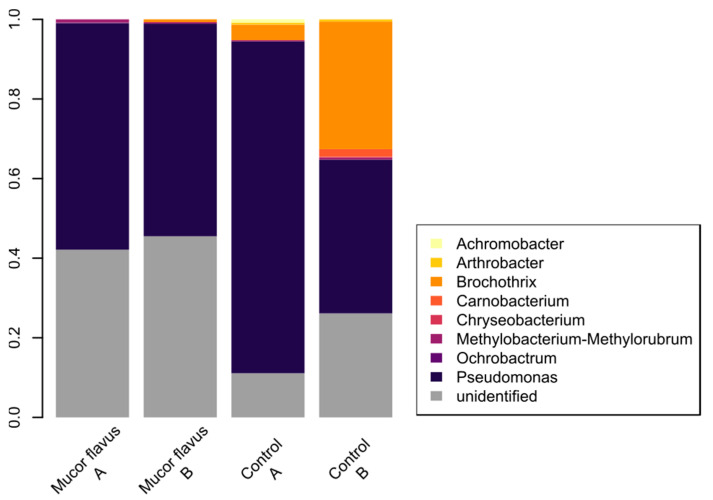
Relative abundance in (%) of bacterial communities at genus level in control samples and in samples inoculated with *M. flavus* biostarter.

*Proteobacteria* and *Firmicutes* were the dominant phyla across the samples. However, unidentified species of bacteria were also abundant. *Firmicutes* representatives were more abundant in control samples (Figure 7). The meat samples aged with the *M. flavus* biostarter were characterized by a similar, stable bacterial community, composed mainly of *Pseudomonas* sp. and some unidentified bacterial taxa, while *Brochothrix* sp. was observed in control samples. The existence of food-borne pathogens, such as *Bacillus cereus*, *Staphylococcus aureus*, *Listeria monocytogenes*, or *Escherichia coli*, was not detected in any sample.

## 4. Discussion

Dry-aged beef (DAB) is recently gaining popularity as it is appreciated for its unique flavor. On the one hand, these evolving aromas may partially derive from the microbial metabolic activity on the meat’s surface, on the other, undesirable microorganisms might cause meat spoilage [8].

However, apart from the impact of microorganisms, one of the critical factors affecting the dry-aging process is the initial meat quality. The raw material pH value should range between 5.4 and 5.7 and the intramuscular fat content should be at least in the range of 6–11% [1,2]. Such parameters guarantee that the final product will have the appropriate flavor, tenderness, and juiciness. Therefore, due to the elevated cost and time-consuming nature of this process, only the highest quality meat should be dry-aged [9]. The meat used in this experiment met these criteria (Table 1) and at the beginning of aging, the values of parameters were similar between the entrecotes untreated and treated with the *M. flavus* biostarter.

Not only the longer time of dry-aging or the dynamic microbial activity influence the high prices of final products. This also results from the decrease in the DAB weight, which is caused by moisture loss [5]. In the presented experiment, the average weight loss after four weeks of aging was approximately 21.4% (Figure 1), with more pronounced losses at the beginning of the process and smaller towards the end. The resulting average daily weight loss of ca. 0.76% was slightly higher than in other published studies where it varied from 0.26 to 0.74% per day [7,9,35]. Similar to the results of Hanagasaki and Asato [14], we did not observe significant differences in weight losses between the controls and the samples aged with the *M. flavus* biostarter. Likewise, the changes in weight losses over time were similar in both experiments. They were higher in the first 3 weeks, followed by a reduction in the last week. Finally, the moisture losses during dry-aging seem to depend primarily on the aging parameters, i.e., temperature, air movement, humidity, and incubation time [5,8,9].

Although in the majority of studied parameters, no significant differences were found between the studied groups, the obtained values were similar to the data presented by Kim et al. [35], Iida et al. [36], and Ribeiro et al. [37]. On the other hand, there was a statistically significant increase in the pH value, from 5.60 to 5.84 (*p* < 0.05), during the aging of meat with the use of the *M. flavus* biostarter. In the control group instead, the increase was smaller and insignificant. According to Terjung et al. [38], the pH increases during the first four weeks of dry-aging and then drops. However, results of various studies are not conclusive. While Kim et al. [34] reported the same tendencies as did Terjung et al. [38], Iida et al. [36] showed no increase in pH during dry maturation of beef. Further, Obuz et al. [39] showed some elevated pH values of DAB, suggesting that this is due to nitrogenous compounds deriving from protein hydrolysis. However, other studies suggested a correlation between the pH value drop and increased lactic acid bacterial growth [40]. Finally, Terjung et al. [38] postulated that the pH level is influenced mainly by the type of the aging process (wet vs. dry) and the explicit impact this parameter, especially in DAB, has on the microbiota of meat. Thus, most probably, the elevated values of pH seen in our study derive from the activity of the *M. flavus* biostarter.

Molds, but also other kinds of fungi, develop better at pH between 5 and 7, which is typical for beef [13]. Therefore, the changes of pH during the aging process may affect the developing microbial communities. Ryu et al. [28] described changes in the DAB microbiome over 160 days, stating that at the beginning lactic acid bacteria (LAB) are particularly common, but their population reduced over time, and they were replaced with other bacterial groups such as *Pseudomonas* sp. [41]. Those bacteria species were also dominant in our samples (Figure 7). On one hand, it was shown that *Pseudomonas* sp. is able to spread along fungal hyphae [42]. This may explain its dominance in samples with the *M. flavus* biostarter. On the other hand, it is known that the representatives of *Mucoromycota* (where *M. flavus* belongs) can form various ecological relationships with different bacterial groups and some of these interactions are even endohyphal [43]. In our experiment, the bacterial community of control samples was more variable than the one formed within samples aged with the *M. flavus* biostarter. This kind of occurrence can be explained by the pH changes, which might have been induced by the fungal presence, although other changes in conditions, such as humidity, cannot be excluded. Fungal mycelium development on meat might serve as a reservoir of water during the dry-aging process, possibly stabilizing bacterial growth.

The observed increase in pH value during the aging of the meat with *M. flavus* not only affected the microbial community diversity but is also particularly important for the further course of the dry-aging process and the physicochemical properties of the final product. Kim et al. [5] stated that the ultimate pH level of muscle tissue plays a critical role in the degradation of myofibrillar proteins. Analysis of the degradation of myosin light chains MLC2 (Figure 3 and Figure 4) using the SDS-PAGE method showed significant differences between studied groups. A higher degree of MLC2 break down was found in beef aged with the *M. flavus* biostarter. According to Picard et al. [44], Sierra et al. [45], and Ding et al. [46], the degree of degradation of those proteins (MLC2) is one of the indicators associated with the beef myofibrillar proteins’ effective proteolysis, particularly related to the tenderness of meat.

It was observed that beef with higher pH had the highest tenderness and myofibril fragmentation index, most probably due to the fact that proteins such as desmin and troponin-T appeared to be more degraded [47]. However, results of advanced proteolysis level and elevated pH values are not transparent, showing no effect of ultimate pH on the tenderness of dry-aged beef [48]. However, the meat aged with the *M. flavus* biostarter had elevated pH values [13] and simultaneously a higher MLC2 protein degradation level, suggesting the intense proteolytic activity of growing fungi, significantly affecting the meat tenderness. A higher degradation rate of myosin and actin while aging with biostarters was also demonstrated by other authors in fermented sausages [49,50]. The proteolytic activity of *M. flavus* and its ability to degrade proteins was additionally proven in soybean fermentation at a low temperature [51].

As mentioned above, the biological and physicochemical properties of the meat changed after *M. flavus* biostarter addition. Most importantly, it had significant positive effects on meat sensory parameters such as odor of meat after grilling, juiciness, softness, and acceptability (Figure 5). Especially, the better juiciness and softness of meat may be connected with the higher pH. This is mainly caused by a better water-holding capacity of muscle proteins in such conditions [5]. Water-holding capacity is often improved in long-term aged meat and is usually linked with postmortem proteolysis of structural proteins [6]. Huff-Lonergan and Lonergan [52] called this the “sponge effect” and proved that degradation of costamere linkages during postmortem aging reduced myofibril shrinkage as well as leaving more space within muscle fibers to retain water. In addition, it has been proposed that myofibrillar proteins are broken down to disturb the drip channel and, hence, increase the ability of the muscle to hold the water within the cell [53]. Overall, this better water-holding capacity translates into the better juiciness and softness of DAB. Many studies showed that dry-aged beef was characterized by better flavor, odor, and overall liking [7,36,54,55]. It is also often considered as having more favorable palatability. The taste of DAB has stronger notes such as beefy/brothy, roast meat, and brown-roasted meat as well as also scoring higher for umami, buttery, caramelized, sweet, and nutty flavors [5,8].

It is known that mold and yeast are involved in the aroma production in DAB. These volatile compounds derive considerably from lipolysis of free fatty acids (FFAs) through lipolysis activity. FFAs play a considerable role in the formation of aroma volatiles including esters [56] and they are one of the main taste-active compounds in meat and meat products [52]. Overall, the aging of meat using microbial biostarters, including the one based on *M. flavus*, may improve the palatability of the meat [14]. It was highlighted that meat tenderized with *M. flavus* has higher amounts of functional sweet-, savory-, and umami-tasting amino acids. Additionally, *M. flavus* is a fungus which naturally occurs on aged meat [14,27,50].

## 5. Conclusions

*M. flavus* can be applied as an inoculum in order to standardize the dry-aging of beef. Here, we showed that DAB inoculated with this biostarter may have an increased pH level, stronger light myosin chain proteolysis rate, and improved sensory quality parameters. The meat aged with the *M. flavus* biostarter is characterized by a stable bacterial community, lacking pathogens with dominating occurrence of *Pseudomonas* sp. Summarizing, the *M. flavus*-based biostarter was shown to increase the quality of DAB. The data of the work are limited in number and certainly this topic needs further investigation.

## 6. Patents

The research solution, describing application of the *Mucor flavus* KKP 2092p for beef dry-aging, has been filed at the Patent Office of the Republic of Poland and has been assigned an application number: P.443722.

## Figures and Tables

**Table 1:** Characteristics of beef quality before dry-aging.

Traits	Group		SEM
	Biostarter−	Biostarter+	
pH	5.61	5.60	0.05
Color parameters: L*	33.03	34.11	1.93
a*	19.32	19.74	1.69
b*	16.50	16.27	1.64
Glucose (mmol/L)	5.83	5.29	0.70
Lactate (mmol/L)	57.24	63.81	5.98
Glycolytic potential (mmol/L)	68.90	74.40	6.75
WHC (cm^2^/g)	13.49	14.00	1.68
Plasticity (cm^2^)	3.37	3.30	0.21
Moisture content (%)	66.84	68.97	1.41
Fat content (%)	9.16	8.80	1.38
Protein content (%)	21.61	20.97	0.51
Connective tissue (%)	0.87	0.84	0.14
Ash content (%)	0.66	0.90	0.14

**Table 2:** Characteristics of beef quality after dry-aging.

Traits	Group		SEM
	Biostarter−	Biostarter+	
pH	5.75	5.84	0.07
Color parameters: L*	28.76	26.63	1.57
a*	25.34	24.61	1.72
b*	24.38	22.67	1.67
Shear force after grilling (N)	75.35	65.91	4.27
Penetration force after grilling (mm)	12.82	12.11	0.55
The content of malondialdehyde (mg/kg)	0.98	0.52	0.43

**Table 3:** The alpha-diversity indices (Shannon, Chao1, and Simpson) of the bacterial communities after 28 d of aging with and without biostarter.

Measure	Biostarter− (Sample 1)	Biostarter− (Sample 2)	Biostarter+ (Sample 1)	Biostarter+ (Sample 2)
Chao1	14	17	10	10
Shannon	0.93	2.25	1.21	1.23
Simpson	0.37	0.86	0.59	0.61

## Data Availability

The sequences obtained in this study were deposited in the NCBI GenBank database. Voucher specimens were deposited in the Herbarium of the Faculty of Biology, University of Warsaw.

## Supplementary Materials

The following supporting information can be downloaded at: https://www.mdpi.com/article/10.3390/foods12061330/s1, Figure S1: Rarefaction plots of the bacterial communities on the surface of dry-aged beef with respect to samples origin (control samples (B−): 1–2, samples inoculated with *M. flavus* biostarter (B+): 3–4. Rarefaction curves display the number of operational taxonomic units (ASVs)) detected based on the sampling intensity of the libraries.
