# Supplementary material for: Fungal Biostarter Effect on the Quality of Dry-Aged Beef
Source: Foods. 2023 Mar 21;12(6):1330. doi: 10.3390/foods12061330 (PMC10048090; doi:10.3390/foods12061330)
Supplement: Supplementary file 1 [file foods-12-01330-s001.zip › foods-2260619-supplementary.pdf]

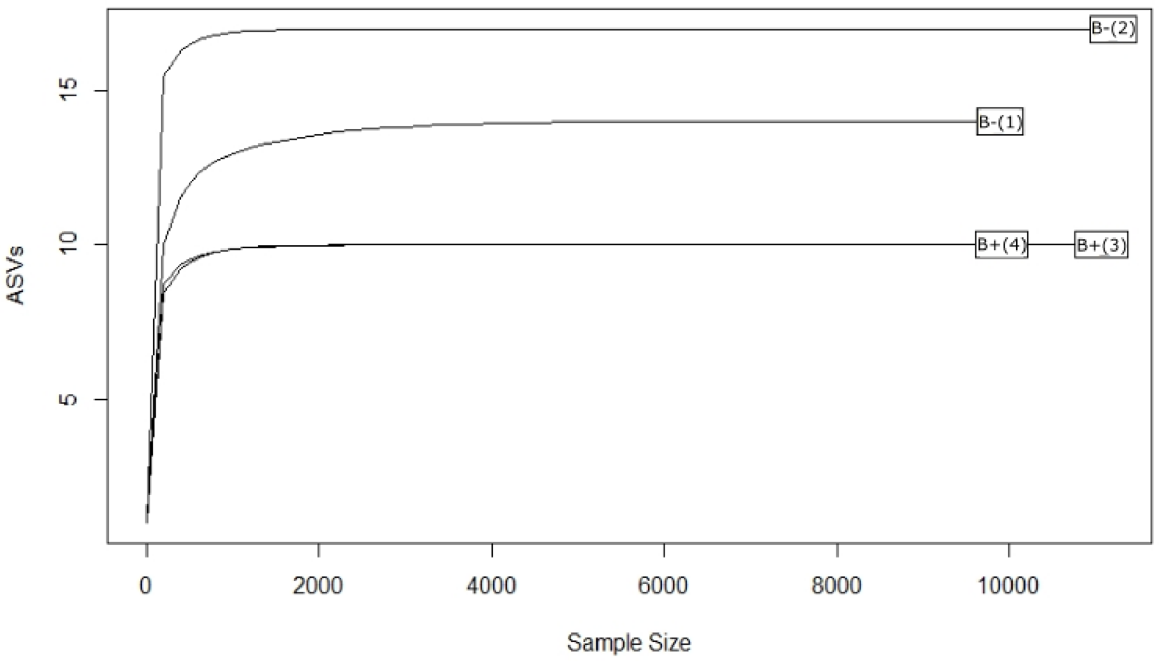

Figure S1. Rarefaction plots of the bacterial communities on the surface of dry-aged beef with respect to samples analyzed (control samples (B-): 1-2, samples inoculated with *M. flavus* biostarter (B+): 3-4. Rarefaction curves display the number of operational taxonomic units (ASVs) detected based on the sampling intensity of the libraries
